# Congenital Giant Dysplastic Kidney Presenting as Respiratory Distress: A Case Report

**DOI:** 10.21699/jns.v6i1.454

**Published:** 2017-01-01

**Authors:** Mahesh Kumar, Geeta Gathwala, KN Ratttan, Sandeep Lather, Poonam Dalal

**Affiliations:** 1 Neonatal Services Unit, Department of Pediatrics PGIMS, Rohtak; 2 Department of Pediatric Surgery, PGIMS Rohtak

**Keywords:** Newborn, Giant, Dysplastic kidney, Respiratory distress

## Abstract

Multicystic dysplastic kidney (MCDK) is the most common form of renal cystic disease in children and is one of the most common causes of abdominal mass in infancy. Here in we are reporting a rare case of a large MCKD that caused respiratory compromise and the infant presented with respiratory distress.

## CASE REPORT

A primigravida with supervised pregnancy delivered a full term 3.7 kg male baby at PGIMS Rohtak. The baby was born of non-consanguineous marriage, with no history of exposure to any teratogens. Baby cried immediately at birth and Apgar score was normal. On examination, baby appeared healthy but had a huge distended abdomen. The infant was in respiratory distress with respiratory rate of 70/min and mild subcostal retractions. Baby was admitted to NICU and started on oxygen via nasal prongs and other supportive management. Nasogastric tube was inserted and anal patency was checked. The Mean arterial pressure was 45 mmHg. The abdomen was distended and a huge cystic abdominal mass occupying whole of the abdomen was palpable. X-ray abdomen showed a huge homogenous soft tissue opacity occupying almost whole abdomen pushing gut to the left side. (Fig. 1) CT scan abdomen showed a large multicystic kidney lesion measuring 14x 11.5x 8.5 mm arising from right renal fossa. The lesion was crossing the midline and displacing bowel loops with no invasion in solid viscera.

Explorative laparotomy revealed a huge multicystic mass in right retroperitoneum arising from right kidney, occupying the whole abdomen. The multicystic mass was excised in toto. There were multiple cysts of variable sizes and the largest cyst measured 16cmx 16cm (Fig.2). On histopathological examination, the diagnosis of right multicystic dysplastic kidney was confirmed. Postoperative period was uneventful. Postoperatively, the respiratory distress settled and the patient was discharged on 10th postop day on full oral feeds. On follow up, he is doing well.

**Figure:**
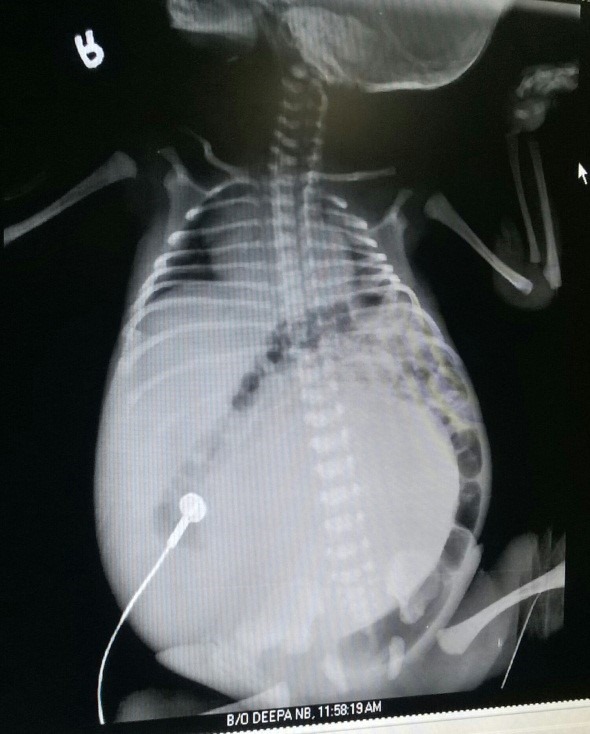
Figure 1: X-ray abdomen showed a huge homogenous soft tissue opacity occupying almost whole abdomen pushing gut to the left side.

**Figure:**
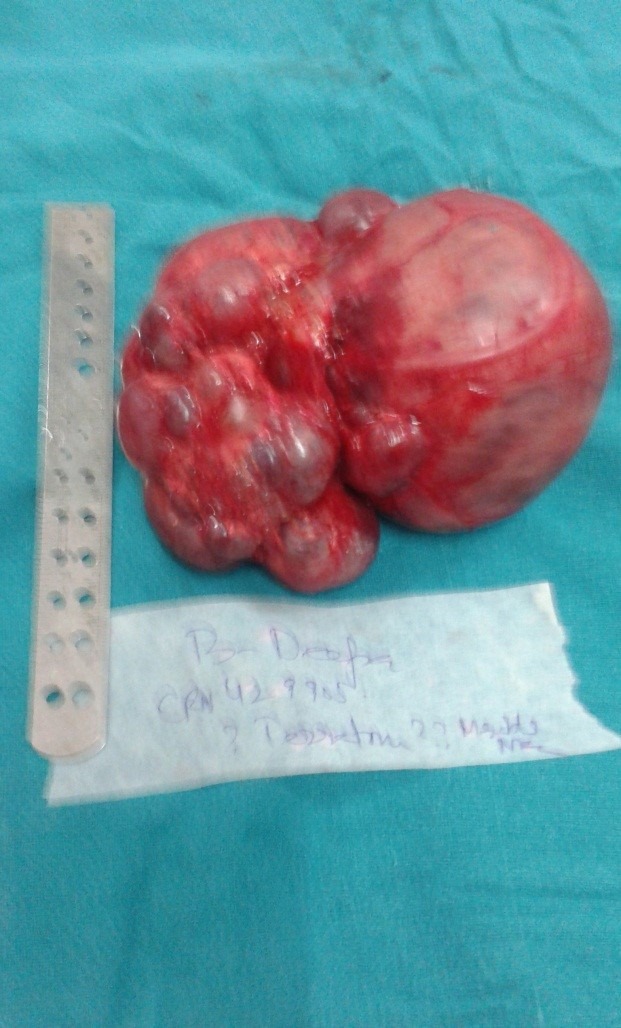
Figure 2: Excised right kidney showing multiple cysts of variable sizes and the largest cyst measured 16cmx 16cm.

## DISCUSSION

MCDK is the most common form of renal cystic disease in children and is one of the most common causes of abdominal mass in infancy. It consists of cysts of varying number and size with small intervening islands of dysplastic parenchymal tissue, including immature glomeruli, primitive tubules and cysts derived from tubular and glomerular structures. The pathogenesis of MCDK is unknown, possibly results from abnormal metanephros differentiation, probably due to disturbed connection of ureteric bud with renal blastema and abnormal division at the stage of metanephros.[1] The left kidney is involved more commonly (55%) than the right kidney (45%). But our index neonate had MCDK on the right side. Also as per literature, males are affected more frequently than females (male: female ratio-1.48:1).[2] Similarly, our index case was also male. Bilateral MCDK, which is often incompatible with life, contributes to 20% of prenatally diagnosed cases. Dysplastic kidney may persist without any change, increase in size, or might undergo spontaneous involution. The initial MCDK length is the most important factor for predicting complete involution.[3] In prenatally diagnosed cases, the abnormal kidney is palpable in only 13–22% of patients. It is usually asymptomatic and can remain undetected into adulthood, if not detected antenatally. But our case presented with huge abdominal mass presenting with respiratory distress which has been reported occasionally.[4] It is important to distinguish MCDK from poorly functioning hydronephrotic kidneys and multilocular cystic renal tumor.[5] ‘Prophylactic’ nephrectomy of MCDK in the 1980s was recommended by some authors although precise indications for removing these kidneys were unclear.[6] According to CUA update 2014, nephrectomy continues to be suitable treatment option for MCDKs that do not involute, or are unlikely to involute.[7] Currently, the indications for resecting the MCDK are restricted to: (i) very large MCDKs that interfere with respiratory or intestinal function in the neonatal phase; and (ii) that contain solid parts that seem to grow during the follow-up on ultrasonography; and (iii) Hypertension. Using these indications, there was a need for surgery in our case. The respiratory distress in the index case settled after the mass was removed.

## Footnotes

**Source of Support:** Nil

**Conflict of Interest:** Nil

## References

[1] Caglayan AO, Gumus H, Erdogan I. A female case with multicystic dysplastic kidney: new findings, genetic counseling, and literature review. Cent Eu J Urol. 2010.151-2.

[2] Robson WL, Leung AK, Thomason MA. Multicystic dysplasia of the kidney. Clin Pediatr. 1995; 34:32–40. 10.1177/000992289503400106 7720326

[3] Dogan CS, Torun-Bayram M, Aybar MD. Unilateral multicystic dysplastic kidney in children. Turk J Pediatr. 2014; 56:75-9. 24827951

[4] Middleton AW Jr, Melzer RB. Neonatal multicystic kidney with associated respiratory distress, obstruction of contralateral ureter and gastric compromise. Urol. 1989; 34:36-8. 10.1016/0090-4295(89)90153-2 2501927

[5] Campagnola S, Fasoli L, Flessati P. Congenital cystic mesoblastic nephroma. Urol Int. 1998; 61254–6. 10.1159/000030342 10364762

[6] Menster M, Mahan J, Koff S. Multicystic dysplastic kidney. Pediatr Nephrol. 1994; 8:113-5. 10.1007/BF00868287 8142209

[7] Poosy K. Multicystic dysplastic kidney (MCDK) in the neonate: The role of the urologist. Can Urol Assoc J. 2016; 10:18-24. 10.5489/cuaj.3520 PMC4771553 26977201

